# Astrocyte Pannexin 1 Suppresses LPS-Induced Inflammatory Responses to Protect Neuronal SH-SY5Y Cells

**DOI:** 10.3389/fncel.2021.710820

**Published:** 2021-08-12

**Authors:** Zhuo-Min Ling, Qian Wang, Yu Ma, Peng Xue, Yun Gu, Mao-Hong Cao, Zhong-Ya Wei

**Affiliations:** ^1^Key Laboratory of Neuroregeneration of Jiangsu and Ministry of Education, Jiangsu Clinical Medicine Center of Tissue Engineering and Nerve Injury Repair, Co-Innovation Center of Neuroregeneration, Nantong University, Nantong, China; ^2^Medical School of Nantong University, Nantong, China; ^3^Department of Neurology, Affiliated Hospital of Nantong University, Nantong, China

**Keywords:** Pannexin 1, astrocytes, neuroinflammation, Parkinson’s disease, MPP^+^

## Abstract

Reactive astrogliosis is a key hallmark of inflammatory responses in the pathogenesis of brain injury, including Parkinson’s disease (PD), but its role and regulatory mechanisms are not fully understood. Pannexin 1 (Panx 1) is a membrane channel that mediates substance release in many neurodegenerative diseases. However, the role of astrocyte Panx 1 in the regulation of PD-like neuroinflammation remains elusive. Here, we characterized the expression of Panx 1 in isolated primary astrocytes and a 1-methyl-4-phenyl-1,2,3,6-tetrahydropyridine (MPTP)-induced PD model. The functions of Panx 1 in inflammatory cytokines expression and the viability of neuronal SH-SY5Y cells were examined in cultured cells treated with lipopolysaccharide (LPS) and 1-methyl-4-phenylpyridinium (MPP^+^). We found that Panx 1 expression was significantly increased under both LPS- and MPP^+^-treated conditions. Panx 1 downregulation suppressed LPS-induced pro-inflammatory cytokine expression but did not significantly affect MPP^+^-induced astrocyte apoptosis or inflammatory cytokine expression through treatment with the Panx 1 inhibitor carbenoxolone (CBX) and Panx 1 siRNA. Moreover, silencing Panx 1 in reactive astrocytes had a potentially protective effect on the viability of neuronal SH-SY5Y cells. Therefore, we propose that Panx 1 may serve as a key regulator in reactive astrocytes to intervene in the inflammatory response and maintain neuronal viability in the context of PD-like conditions.

## Introduction

Parkinson’s disease (PD) is characterized by damage to the ascending nigrostriatal pathway, including progressive loss of dopaminergic (DA) neurons in the substantia nigra (SN) and axon terminals in the striatum. Therefore, most previous studies have focused on the degeneration of DA neurons, but the mechanism is still far from clear. Of interest, emerging evidence has revealed the regulative functions of glial cells in the initiation, development and treatment of PD (Booth et al., 2017; Yun et al., 2018; Domingues et al., 2020; Kam et al., 2020). Astrocytes, which constitute the most abundant glial cell type in the central nervous system, play a key role in maintaining homeostasis under physiological conditions by releasing a variety of signaling molecules and regulating blood flow and synaptic transmission (Wei and Morrison, 2020). However, during the onset and progression of PD, astrocytes are converted into reactive astrocytes; during this process, the astrocytes undergo proliferation, hypertrophy and produce a variety of potential neuroprotective or toxic mediators, which are neurotoxic or neuroprotective to the surrounding DA neurons (Booth et al., 2017; Acioglu et al., 2021). Therefore, under both physiological and pathological conditions, astrocytes contribute to the functions of DA neurons.

Lipopolysaccharide (LPS) and 1-methyl-4-phenyl-1,2,3,6-tetrahydropyridine (MPTP) are widely used to produce animal models of PD (Jackson-Lewis and Przedborski, 2007; Meredith et al., 2008). LPS, an endotoxin is often used for both *in vivo* and *in vitro* PD model to study the mechanism of the neuroinflammation, and its toxic effect is due to the dopaminergic vulnerability to the substantia nigra (Herrera et al., 2000; Gao et al., 2011; Wang et al., 2016; Zhang et al., 2020). As for MPTP, 1-methyl-4-phenylpyridinium (MPP^+^) is the ultimate mediator of the neurotoxicity of MPTP. Once MPTP targets the brain, it is successively metabolized to MPP^+^ by the enzyme monoamine oxidase type B, and it retains in catecholaminergic terminals via monoaminergic transporters (Johannessen, 1991). This enzyme is not found in DA neurons, and is located mainly in glial cells (Levitt et al., 1982; Westlund et al., 1985). Moreover, astrocytes are primarily responsible for converting MPTP into the active toxic compound MPP^+^ (Ransom et al., 1987; Di Monte et al., 1992). However, the effects of extracellular MPP^+^ on surrounding astrocytes are not fully understood.

Gap junctions are important pathways not only for communication between neighboring cells but also for signaling transmission between the intracellular and extracellular compartments (Oshima, 2020). Many previous studies have uncovered the potential role of astrocyte gap junctions in PD regulation, but most of them have focused on connexins (Freitas-Andrade and Naus, 2016; Ahmadian et al., 2019). Of note, modulation of astrocyte connexins 43 and 30 (CX 43 and CX 30, respectively) has been revealed to be a key element in the regulation of PD pathogenesis (Kawasaki et al., 2009; Fujita et al., 2018). However, there are very few data regarding the roles of astrocyte pannexins in PD-like conditions. The pannexin family primarily includes three main members, Panx 1, Panx 2, and Panx 3, which are widely expressed in neurons and glial cells, and respond to various activation and mediate both non-selective ion permeability and signaling molecules release (Bruzzone et al., 2003; Yeung et al., 2020). Although fibroblast growth factor 1 (FGF1) has been found to induce excess ATP release through CX 43 and Pannexin 1 (Panx 1) in spinal cord astrocytes, resulting in production of inflammatory cytokines that damage to neighboring neurons (Garre et al., 2010, 2016; Freitas-Andrade and Naus, 2016); and the activation of the inflammasome by high extracellular potassium may be mediated by Panx 1 in cultured astrocytes (Silverman et al., 2009), the roles of astrocyte pannexins in the context of neuroinflammation, especially both in LPS and MPP^+^ stimuli are not fully understood.

Here, we tested the expression of Panx 1 in primary cultured astrocytes and brain slices and confirmed that Panx 1 was expressed primarily in astrocytes. To investigate the role of astrocyte Panx 1 for inflammatory response, we used LPS and MPP^+^ to mimic the conditions of PD neuroinflammation and apoptosis in this study. The data suggest that Panx 1 is required for LPS-induced inflammatory responses but not for MPP^+^-induced apoptosis. Moreover, the ameliorative effect of Panx 1 on LPS-induced neuroinflammation is beneficial for the viability of neuronal SH-SY5Y cells.

## Results

### Characterization of Pannexin 1 Expression in Astrocytes

**FIGURE 1:**
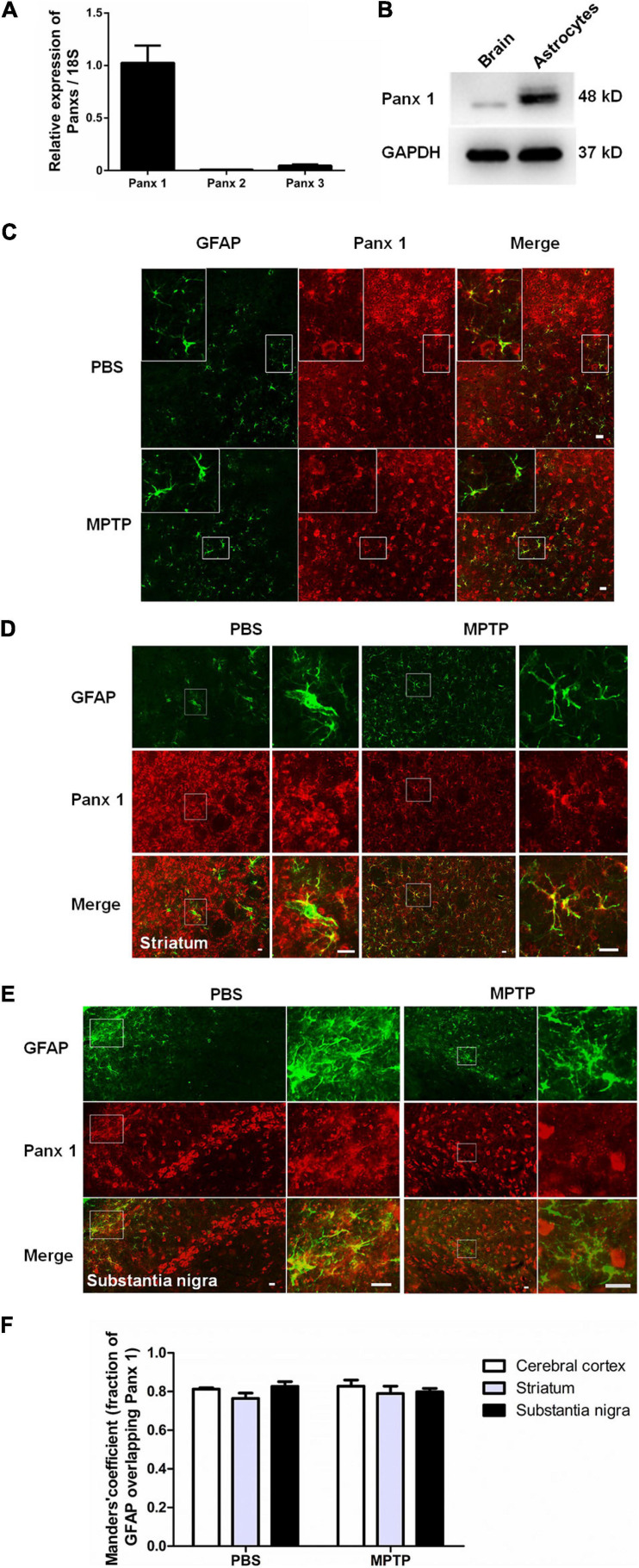
Characterization of Panx 1 expression in astrocytes. **(A)** qPCR analysis of Panx 1, Panx 2, and Panx 3 mRNA levels in cultured primary astrocytes normalized to 18S mRNA levels. **(B)** Panx 1 protein levels in cultured primary astrocytes was measured by Western Blotting and the brain was used as a positive control. **(C–E)** Panx 1 immunostaining in the cerebral cortex, striatum and SN in wild type mice after injection with PBS or MPTP on 4 days. Enlarged images are from the inset box. The sections were double-stained with Panx 1 (red) and GFAP (green). **(F)** Manders’ coefficient of the fraction of GFAP overlapping Panx 1 in the cerebral cortex, striatum and SN. Scale bar, 20 μm.

As expected given previous results showing that pannexins are widely expressed in primary cultured astrocytes (Wei et al., 2016; Scemes et al., 2019), we found that Panx 1 was more highly expressed in primary cultured astrocytes than Panx 2 and Panx 3 at the mRNA level (Figure 1A). Next, we utilized western blotting to further confirm Panx 1 expression in astrocytes, and the results showed that a single band of Panx 1 was detectable in primary cultured astrocytes (Figure 1B). Emerging evidence has showed that reactive astrocytes are key elements in the inflammatory responses of PD (Niranjan, 2014; Booth et al., 2017; Rizor et al., 2019). To investigate the expression of Panx 1 in reactive astrocytes in this process, we estimated the expression of astrocyte Panx 1 in an acute PD model with reduced doses of MPTP systemic administration (Jackson-Lewis and Przedborski, 2007; Meng et al., 2019). MPTP triggered obvious loss of DA neurons in the SN; activated astrocyte proliferation in the striatum (Supplementary Figure 1), and caused astrocyte hypertrophy in the cerebral cortex, striatum, and SN (Figures 1C–E). Immunostaining for GFAP and Panx 1 in astrocytes in the cerebral cortex, striatum and SN showed a robust co-localization (about 80%) in both the PBS-treated and MPTP-treated groups (Figure 1F). Taken together, these data suggest that Panx 1 is expressed in both primary cultured astrocytes and reactive astrocytes in MPTP-treated mice.

### Silencing Panx 1 Does Not Affect Inflammatory Cytokine Production or Apoptosis in MPP^+^-Treated Astrocytes

**FIGURE 2:**
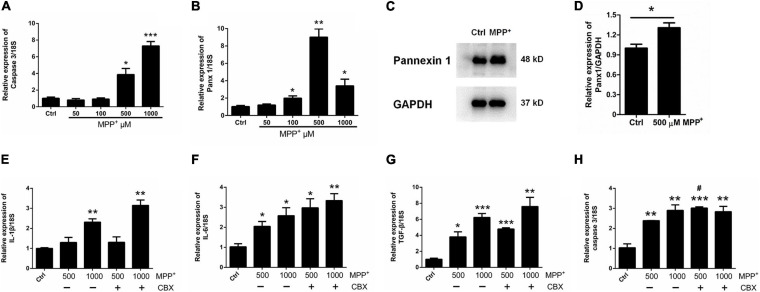
Effect of CBX on MPP^+^-induced apoptosis in cultured astrocytes. **(A,B)** The effect of different doses of MPP^+^ (0, 50, 100, 500, and 1,000 μM) on the expression of caspase 3 and Panx 1 by qPCR assay. **(C,D)** western blotting analysis of Panx 1 expression after treatment with 500 μM MPP^+^. Protein levels were quantified and normalized to the GAPDH levels. **(E–H)** Influence of CBX (100 μM) on the expression of caspase 3 and selected inflammatory cytokines IL-1β, IL-6, and TGF-β in MPP^+^-induced astrocyte apoptosis. The data were normalized to 18S mRNA levels, *n* = 3. All data are mean ± SEM. Statistical comparison for **(A,B,E–H)** were performed using one-way ANOVA, following by Newman–Keuls Multiple Comparison Test, and for **(D)** using Student’s *t*-test. *p < 0.05, **p < 0.01, ***p < 0.001 compared with control group (0 μM MPP^+^, ^#^*p* < 0.05 compared with the group of 500 μM MPP^+^ alone).

Considering the fact that human neuroblastoma SH-SY5Y cells (a widely used cellular model to study PD) are able to express a lot of features characteristic for dopaminergic neurons, including dopamine-β-hydroxylase and tyrosine hydroxylase activities (Kovalevich et al., 2021), we chose SH-SY5Y cells as a positive control to test the toxicity of MPP^+^. We first treated SH-SY5Y cells with MPP^+^ at doses of 50, 100, 500, and 1,000 μM for 24 h. A CCK-8 assay showed that cell viability declined with increasing dose (Supplementary Figure 2A). However, when primary cultured astrocytes were treated with 50, 100, 500, or 1,000 μM MPP^+^ for 24 h, the results showed that MPP^+^ had a toxic effect on cell viability only at 500 and 1,000 μM (Supplementary Figure 2B); in addition, it significantly increased the expression of selected inflammatory cytokines interleukin-1β (IL-1β), interleukin-6 (IL-6), and transforming growth factor-β (TGF-β) (Supplementary Figures 3A–C). Furthermore, caspase 3 mRNA levels were significantly increased by 500 and 1000 μM MPP^+^ treatment (Figure 2A). These data suggest that MPP^+^ exerts toxic effects on astrocytes at elevated-concentrations.

**FIGURE 3:**
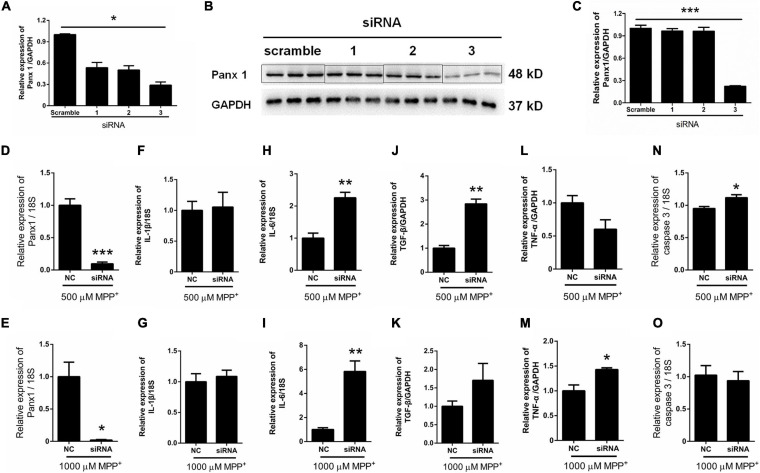
Effect of Panx 1 downregulation on MPP^+^-induced apoptosis in primary cultured astrocytes. **(A–C)** The silencing efficiency of three Panx 1-targeting siRNA was determined by qPCR and western blot analysis in primary cultured astrocytes. **(D,E)** qPCR analysis of Panx 1 expression in negative control and siRNA groups treated with 500 or 1,000 μM MPP^+^. **(F–M)** Effect of Panx 1 downregulation on the expression of selected inflammatory cytokines, including IL-1β, IL-6, TNF-α, and TGF-β in MPP^+^-induced astrocytes. **(N,O)** Influence of Panx 1 siRNA on caspase 3 expression in MPP^+^- induced astrocytes. The data were normalized to GAPDH or 18S mRNA levels, *n* = 3. All data are mean ± SEM. Statistical comparison for **(A,C)** were performed using one-way ANOVA, following by Newman–Keuls Multiple Comparison Test, and for **(D–O)** using Student’s *t*-test. *p < 0.05, **p < 0.01, ***p < 0.001 compared with negative group.

Surprisingly, as illustrated in Figure 2B, the expression of Panx 1 at mRNA level was significantly increased under treatment with MPP^+^ at concentrations above 100 μM. Moreover, we observed a significant increase of Panx 1 expression at the protein level (up by 30.6%) when compared with its baseline level (Figures 2C,D). Next, we evaluated the role of Panx 1 upregulation in MPP^+^-evoked astrocyte apoptosis. MPP^+^ at 500 and 1,000 μM significantly elevated the expression of cytokines, including IL-1β, IL-6, and TGF-β, in primary cultured astrocytes compared with the baseline levels (Figures 2E–G). However, these increase was not abolished by pretreatment with CBX, a Panx 1 inhibitor (100 μM) for 1 h (Figures 2E–G). We also found that CBX did not attenuate caspase 3 expression in MPP^+^-treated astrocytes (Figure 2H). We further confirmed the role of Panx 1 in MPP^+^-treated astrocytes by treating cells with Panx 1 siRNA. We initially screened siRNAs and selected the most effective Panx 1 siRNA in primary cultured astrocytes by qPCR and western blotting analysis (Figures 3A–C). In addition, we screened the effects of the selected siRNA on the expression of Panx 2 and Panx 3. No significant increases in Panx 2 and Panx 3 mRNA expression were detected in astrocytes (Supplementary Figure 4). Under conditions of 500 and 1,000 μM MPP^+^-treatment, Panx 1 expression in astrocytes was significantly attenuated by Panx 1 siRNA (Figures 3D,E, down by 90.5% and 98.6% separately). However, MPP^+^-induced IL-1β, IL-6, tumor necrosis factor-α (TNF-α), and TGF-β expression was not attenuated by pretreatment of cells with Panx 1 siRNA (Figures 3F–M), and Panx 1 siRNA pretreatment also had a relatively little effect on the expression of caspase 3 (Figures 3N,O). As to exclude the side effect of Panx 1 siRNA on the expression of inflammatory factors, we selectively detected the expression of IL-1β, IL-6, and TNF-α after treatment with NC and Panx 1 siRNA alone. The results showed that without MPP^+^-stimuli, there is no significant change between two groups in the expression of inflammatory factors (Supplementary Figure 5). Taken together, these data reveal that Panx 1 has minimal role in MPP^+^-evoked astrocyte apoptosis and cytokine expression.

### LPS Increases Panx 1 Expression in Primary Cultured Astrocytes

**FIGURE 4:**
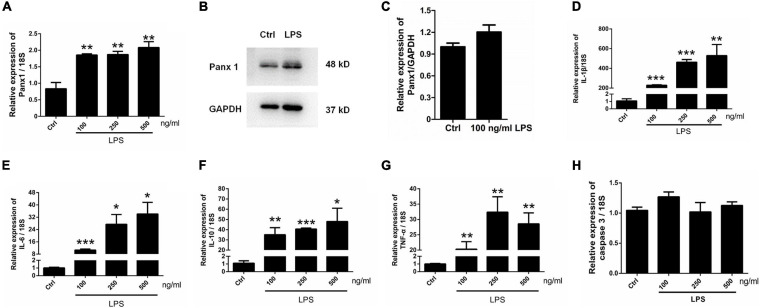
Panx 1 and inflammatory cytokine expression in LPS-treated astrocytes. mRNA expression of Panx 1 **(A)** and the selected inflammatory cytokine IL-1β **(D)**, IL-6 **(E)**, IL-10 **(F)**, TNF-α **(G)** and caspase 3 **(H)** were measured by qPCR assay. Primary cultured astrocytes were treated with LPS at different doses (100, 250, and 500 ng/ml) for 24 h. The levels were displayed as percentage of the control (the same volume of DMSO). The data were normalized to 18S mRNA levels. Western blot analysis of Panx 1 expression **(B,C)** after treatment with 100 ng/ml LPS. Protein levels were quantified and normalized to GAPDH levels, *n* = 3. All data are mean ± SEM. Statistical comparison for **(A,D–H)** were performed using one-way ANOVA, following by Newman–Keuls Multiple Comparison Test, and for **(C)** using Student’s *t*-test. *p < 0.05, **p < 0.01, ***p < 0.001 compared with the control group. Student’s *t*-test.

To further investigate the role of astrocyte Panx 1 in the response to inflammatory stimuli, we treated cells with LPS at 100, 250, or 500 ng/ml for 24 h. The results showed that LPS induced a significant increase at the mRNA level in Panx 1 expression compared with the baseline level but not in a dose-dependent manner (Figure 4A). In addition, a slight increase appeared at the protein level in the expression of Panx 1 (up by 20.4%) after treatment (Figures 4B,C). In contrast, 100 ng/ml LPS treatment did not significantly increase Panx 2 or Panx 3 levels (Supplementary Figure 6). We also found that LPS significantly increase IL-1β, IL-6, IL-10, and TNF-α mRNA expression at all doses (Figures 4D–G). To exclude the possibility that apoptosis was induced, caspase 3 mRNA expression was detected. The data showed that LPS did not significantly elevate caspase 3 levels in astrocytes (Figure 4H). Therefore, we chose 100 ng/ml LPS as an effective dose for the next study.

### Carbenoxolone (CBX) Inhibits LPS-Evoked Inflammatory Cytokine Expression in Primary Cultured Astrocytes

**FIGURE 5:**
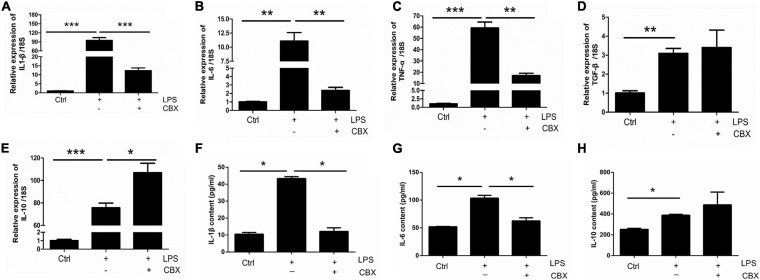
CBX inhibits LPS-induced inflammatory cytokine expression in primary cultured astrocytes. Analysis of the selected inflammatory cytokine mRNA expression by qPCR assay, including IL-1β **(A)**, IL-6 **(B)**, TNF-α **(C)**, TGF-β **(D)**, and IL-10 **(E)** in primary cultured astrocytes. Cells were pretreated with Panx 1 inhibitor CBX (100 mM, 60 min) before LPS treatment (100 ng/ml, 24 h) or be treated with CBX alone. The levels were displayed as percentage of the control (the same volume of DMSO). The data were normalized to 18S mRNA levels. **(F–H)** Evoked expression (content) of IL-1β, IL-6, and IL-10 in astrocytes following with different stimulation, *n* = 3. All data are mean ± SEM. Statistical comparison for were performed using one-way ANOVA, following by Newman–Keuls Multiple Comparison Test. *p < 0.05, **p < 0.01, ***p < 0.001 compared with the control group.

To investigate the role of Panx 1 in LPS-evoked inflammatory cytokine expression, we pretreated primary cultured astrocytes with CBX for 1 h at 100 μM. We first examined the expression of pro-inflammatory and anti-inflammatory molecules in different groups by qPCR, which revealed that the increases in the expression of the pro-inflammatory molecules IL-1β, IL-6, and TNF-α, but not for TGF-β, were significantly attenuated by pretreatment with CBX (Figures 5A–D). Similarly, the LPS-treated group exhibited elevated expression of the anti-inflammatory molecule IL-10, but CBX- pretreated + LPS-treated group exhibited significantly higher expression than the LPS-treated group (Figure 5E). In addition, we selectively detected the cytosolic content of IL-1β, IL-6, and IL-10. Similarly, the increase of IL-1β, IL-6 was blunted by pretreatment with CBX, but no significant increase of IL-10 (Figure 5F–H, LPS/LPS + CBX, IL-1β: down by 75%; IL-6: down by 16.8%). To exclude the effect of CBX on the expression of inflammation-related cytokines, we detected the expression of pro-inflammatory and anti-inflammatory molecules under the same conditions. The results showed that CBX-treatment alone did not affect the expression of these cytokine compared with the baseline levels (Supplementary Figure 7). Collectively, these data suggest that inhibition of Panx 1 with CBX protects against the LPS response by reducing the expression of pro-inflammatory molecules and increasing the levels of anti-inflammatory molecules.

### Panx 1 siRNA Inhibits LPS-Induced Inflammatory Cytokine Expression in Primary Cultured Astrocytes

**FIGURE 6:**
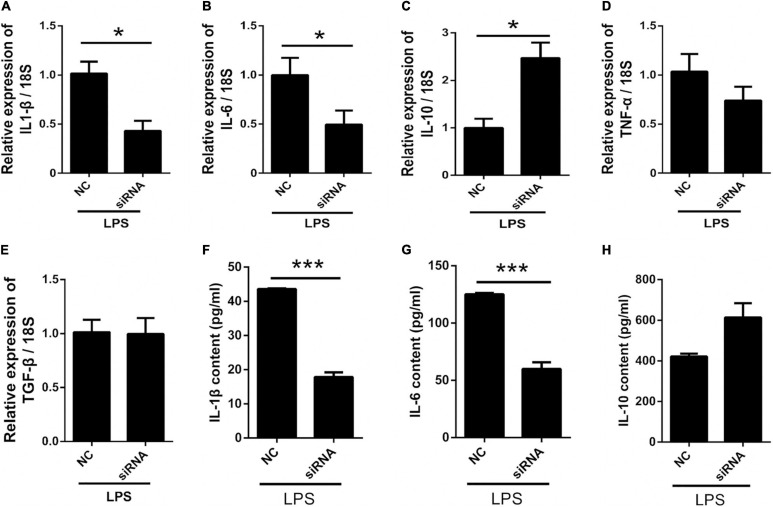
Silencing Panx 1 inhibits LPS-induced inflammatory cytokine expression in primary cultured astrocytes. **(A–E)** The effect of Panx 1 downregulation on LPS-induced inflammatory cytokine mRNA expression, including IL-1β, IL-6, TNF-α, TGF-β, and IL-10 in primary cultured astrocytes by qPCR assay. The data were normalized to GAPDH or 18S mRNA levels. **(F–H)** Evoked expression (content) of IL-1β, IL-6, and IL-10 in astrocytes following with different stimulation, *n* = 3. All data are mean ± SEM. Statistical comparison for were performed using Student’s *t*-test. *p < 0.05, ***p < 0.001 compared with negative control siRNA group.

To further investigate the role of astrocyte Panx 1 in LPS-evoked cytokine expression, we used Panx 1 siRNA to knock down Panx 1 expression in primary cultured astrocytes. Compared with the scrambled siRNA group, the Panx 1 siRNA group exhibited reduced IL-1β and IL-6 levels upon LPS treatment (Figures 6A,B, IL-1β: down by 58.2%; IL-6: down by 50%). Moreover, the expression of IL-10 was significantly increased after knockdown with Panx 1 siRNA (Figure 6C, up by 147%). However, Panx 1 knockdown did not significantly affect the expression of TNF-α or TGF-β (Figures 6D,E). In addition, we selectively detected the cytosolic content of IL-1β, IL-6, and IL-10. Similarly, the increase of IL-1β, IL-6 was blunted by pretreatment with Panx 1 siRNA, but no significant increase of IL-10 (Figures 6F–H, IL-1β: down by 59.1%; IL-6: down by 52.1%). Taken together, these results further illustrate that silencing Panx 1 decreases inflammatory injury.

### Effect of Astrocyte Conditioned Medium on SH-SY5Y Cell Viability

**FIGURE 7:**
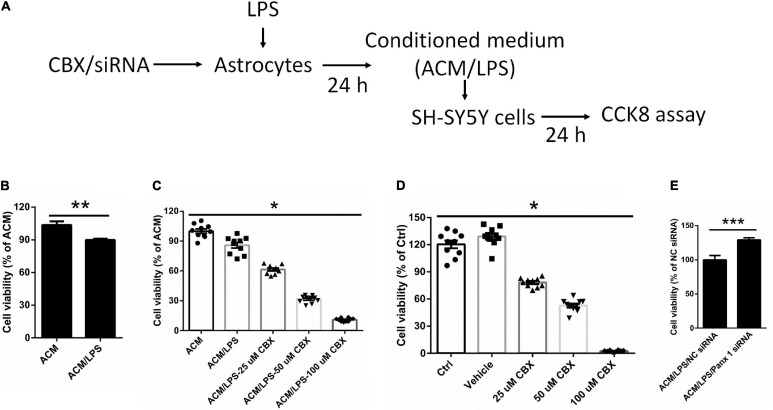
Effect of different conditioned medium on the viability of SY-SY5Y cells. **(A)** Illustration of the experimental design. After treatment with CBX or siRNA, astrocytes were incubated with 100 ng/ml LPS for 24 h. The medium we collected as ACM/LPS were added into SH-SY5Y cells for 24 h and CCK 8 assay was performed to estimate cell viability. **(B)** Impact of ACM/LPS on the viability of SH-SY5Y cells compared with the group of astrocyte conditioned medium + LPS *per se* (without treating astrocytes). **(C)** SH-SY5Y cell viability was assayed by CCK8 assay under different conditioned medium, which from cells pretreated with CBX at different doses before incubation with LPS in astrocytes. **(D)** SH-SY5Y cells were treated with CBX alone at different doses and the viability was measured by CCK8 assay. **(E)** The medium from astrocytes treated with Panx 1 siRNA + LPS were added into SH-SY5Y cells and cell viability was assayed by CCK8 assay. The data were normalized to ACM group levels (ACM + LPS *per se*), *n* = 3. All data are mean ± SEM. Statistical comparison for **(C,D)** were performed using one-way ANOVA, following by Newman–Keuls Multiple Comparison Test, and for **(B,E)** using Student’s *t*-test. *p < 0.05, **p < 0.01, ***p < 0.001 compared with ACM group.

To investigate whether neuronal survival is affected by activation of astrocytes, we cultured SH-SY5Y cells with LPS-activated astrocyte medium and astrocyte conditioned medium + LPS *per se* (without treating astrocytes) (Figure 7A). The results showed that LPS-activated astrocyte medium exerted a negative impact on SH-SY5Y cell viability compared with the control (LPS) medium group (Figure 7B, down by 13.7%).

We further investigated whether silencing astrocyte Panx 1 have an effect on SH-SY5Y cell viability. Conditioned medium collected from CBX preincubated and LPS-activated astrocytes (ACM/LPS-CBX) was added to SH-SY5Y cells. Surprisingly, cell viability was declined with among SH-SY5Y cells pretreated with medium from ACM/LPS-CBX containing CBX at different doses (25, 50, and 100 μM) (Figure 7C). In addition, addition of CBX alone (without treating astrocytes) at 25, 50, and 100 μM negatively affected SH-SY5Y cell viability (Figure 7D). However, compared with LPS treatment, exposure to CBX alone at 100 μM did not induce high levels of inflammatory factors in astrocytes (Supplementary Figure 7). SH-SY5Y cells were also incubated in medium collected from Panx 1- and scrambled siRNA-transfected LPS-activated astrocytes. Cell viability was significantly higher among SH-SY5Y cells-incubated with medium from Panx 1-pretreated cells than among cells incubated with medium from scrambled siRNA-pretreated cells (Figure 7E, up by 29.2%). Collectively, these data reveal that silencing Panx 1 in astrocytes contributes to SH-SY5Y cell viability.

## Discussion

In this study, we investigated the role of astrocyte Panx 1 in inflammatory responses under the conditions of LPS stimulation to establish a model of inflammatory response, and MPP^+^ to induce astrocyte apoptosis. Additionally, we examined whether Panx 1 expression in astrocytes affects neuronal SH-SY5Y cell viability. Our present data suggest that Panx 1 is required for LPS treatment-induced neuroinflammation but not for MPP^+^-induced astrocyte apoptosis. Silencing astrocyte Panx 1 had a potentially protective effect on neuronal SH-SY5Y cell viability.

The death of dopaminergic neurons and reactive astrocytes have been well characterized during the pathological process of PD (Sofroniew and Vinters, 2010; Booth et al., 2017). In our MPTP-induced acute PD model, we also detected histopathological changes, including TH-positive neurons loss in the substantia nigra, and pronounced upregulation of GFAP, cell body hypertrophy and proliferation in different brain areas, especially in the striatum. Of interest, MPTP-induced proliferating astrocytes express Panx 1. Panx 1, a member of the pannexin family, can be opened by many factors, such as extracellular ATP and inflammatory cytokines (Ahmadian et al., 2019). Opening of Panx 1 mediates release of the pro-inflammatory cytokine IL-1β from activated macrophages (Pelegrin and Surprenant, 2006). Thus, reactive astrocyte Panx 1 may contribute to the release of inflammatory cytokines and the regulation of inflammatory responses in PD. However, owing to the lack of astrocyte Panx 1 conditional knock-out mice, we did not perform a lot of *in vivo* experiments to specifically investigate the role of astrocyte Panx 1 in MPTP-induced PD models.

1-methyl-4-phenyl-1,2,3,6-tetrahydropyridine, a widely used parkinsonism-inducing neurotoxin, is taken up and converted into its active metabolite, MPP^+^, in astrocytes (Ransom et al., 1987; Di Monte et al., 1992). Besides its toxic to DA neurons, MPP^+^ also has a direct toxic effect on astrocytes, such as oxidative stress, apoptosis and glutamate homeostasis (Hu et al., 2005; Hua et al., 2019; Rizor et al., 2019). In agreement with the previous studies, we also found that MPP^+^ had a toxic to the primary astrocytes and induced inflammatory responses and caspase 3 activation. Furthermore, we found that MPP^+^-treatment was just able to increase IL-6 and TGF-β expression at lower doses (below 100 μM), but no effect on the caspase 3 expression. However, treatment with 500 μM MPP^+^ for 24 h induced astrocyte apoptosis and pro-inflammatory cytokine production. In addition, there are some differences in the inflammatory factors expression between treatment with 500 and 1,000 μM MPP^+^. Therefore, it is reasonable to indicate that different dose of MPP^+^ induced different cell states and responses. Similarly, glutamate neurotoxicity occurs at low glutamate concentrations, but concentrations of glutamate one-hundred fold higher are required to produce equivalent neurotoxicity to astrocytes (Rosenberg and Aizenman, 1989; Aschner and Kimelberg, 1991). Nevertheless, inhibition of Panx 1 expression or activity in MPP^+^-treated astrocytes was unable to protect astrocytes against pro-inflammatory cytokine production and apoptosis.

Lipopolysaccharide alone is unable to induce astrocyte apoptosis (Sun et al., 2016). This point is supported by our present finding that there were no significant changes in caspase 3 mRNA levels from the baseline levels in astrocytes treated with LPS at different doses. However, it is widely utilized in neuroscience research to generate *in vitro* inflammation models. Accumulating evidence has shown that LPS can trigger astrocytes to release pro-inflammatory cytokines, which damage to the surrounding neurons (Sun et al., 2016; Zhang et al., 2020). Consistently, we observed significant increases in the expression of inflammatory factors, including IL-1β, IL-6, IL-10, and TNF-α, in reactive astrocytes following stimulation with LPS. Panx 1 upregulation was induced by LPS treatment at 100 ng/ml. CBX, a well-known gap junction inhibitor, has shown potential efficacy in improving the pathological conditions of PD (Thakur and Nehru, 2015). For example, CBX has been found to attenuate beta oscillations and improve forelimb function in a PD rat model (Phookan et al., 2015). Moreover, CBX has been found to improve hallmark features via heat shock protein upregulation in a rotenone-based PD model (Thakur and Nehru, 2014). These inhibitory effects of CBX are primarily due to closure of CX 43 gap junctions and Panx 1 channels (Thakur and Nehru, 2015). Our present results confirm that CBX can reduce LPS-induced inflammatory cytokine expression in activated astrocytes. However, CBX is not a specific for inhibitor for pannexin channels; it may also affect connexins (Chen et al., 2014). Moreover, previous studies have demonstrated that modulation of astrocyte CX 43 and CX 30 proteins might play an important role in PD pathology (Kawasaki et al., 2009; Fujita et al., 2018). Therefore, we further tested the role of Panx 1 with Panx 1-targeting siRNA in LPS-induced activated astrocytes and found that knockdown of Panx 1 reduced the expression of pro-inflammatory cytokines and increased the expression of the anti-inflammatory cytokine IL-10. Moreover, we found that silencing Panx 1 alone has no effect on the expression of other pannexin members (Panx 2 and 3), and IL-6, IL-10, and TNF-α expressions. However, in human malignant glioma cells, the levels of IL-6, IL-8, and glutamate were lower in Panx-1 siRNA transfected cells (Wei et al., 2016). Thus, the data we obtained here suggest that Panx 1 is required for inflammatory cytokine expression under LPS-induced inflammatory conditions but not for MPP^+^-induced astrocyte apoptosis. However, the molecular mechanisms of Panx 1 driving the production of these inflammatory factors are not fully understood. In human endothelial cells, Panx 1 involvement in TNF-α-induced IL-1β production through an NF-κB dependent mechanism (Sanchez Arias et al., 2020; Yang et al., 2020). In addition, in early brain injury after subarachnoid hemorrhage, inflammatory response mediated by Panx 1 was primarily through the TLR2/TLR4/NF-κB-mediated signaling pathway (Wu et al., 2017). These data suggest that NF-κB-mediated pathway signaling could be a potential candidate in the regulation of Panx 1-mediated neuroinflammation.

Neuroinflammation, including the inflammatory state of astrocytes, is a key element of PD pathophysiology, and the downstream response of neuroinflammation leads to the death of DA neurons (Booth et al., 2017). Upregulation of Panx 1 in LPS-induced inflammatory response clearly had a negative effect on the viability of SH-SY5Y DA cells in the current study. Downregulation of Panx 1 expression in astrocytes significantly enhanced the viability of SH-SY5Y DA cells under LPS treatment conditions by reducing inflammatory cytokine expression. In addition to glial cells, Panx 1 is also expressed in neurons (Yeung et al., 2020); thus, although CBX can attenuate the release of inflammatory cytokines in astrocytes, it leads to significant death of SH-SY5Y cells in a dose-dependent manner. However, CBX alone treated primary astrocytes, there was no effect on the expression of inflammatory factors. Thus, the side effect of CBX on the surrounding cells needs to be considered when it is used for the treatment of inflammatory disorders.

Accumulating evidence has revealed the role of astrocytes in the regulation of PD. However, the role of reactive astrocytes is still controversial. This controversy might be that astrocytes are neurotoxic or neuroprotective according to the surrounding environment and the nature of the activity signals. In addition to producing potentially toxic materials and killing surrounding neurons (Pekny and Nilsson, 2005; Li et al., 2019), reactive astrocytes can release various neurotrophins, such as glutamate, ciliary neurotrophic factor (Nam et al., 2015) to support the survival and functional recovery of neighboring neurons (Pekny et al., 2014). Thus, in future studies, we will investigate the role of astrocyte Panx 1 in release of neurotrophic factors, especially glial-derived neurotrophic factor (GDNF), and FGF (Lin et al., 1993; Garre et al., 2010, 2016). Although we observed reactive astrocytes in different regions, including the cortex, striatum and SN after MPTP injection in mice, it is difficult to exclude the effects of subregional astrocyte differences on selective neurodegeneration and protection (Zhang and Barres, 2010; Schitine et al., 2015; Kostuk et al., 2019; Matias et al., 2019; Yang and Jackson, 2019). Thus, to elucidate the role of astrocyte Panx 1 in PD, subregional heterogeneity needs to be considered in addition to the secreted molecules. Of note, given with the ubiquitous expression of Panx 1, the role of Panx 1-mediated neuroinflammation could be implicated in many of other disease (Saez et al., 2015; Scemes et al., 2019; Spray and Hanani, 2019; Sanchez Arias et al., 2020) and therefore could represent a potential therapeutic target.

## Conclusion

In this study, we identified the role of astrocyte Panx 1 in PD-like conditions. Upregulation of Panx 1 was observed in LPS- or MPP^+^-induced reactive astrocytes. Astrocyte Panx 1 was required for LPS-induced inflammatory response but not for MPP^+^-induced apoptosis of reactive astrocytes. Importantly, downregulation of astrocyte Panx 1 under LPS induction conditions was beneficial for the viability of SH-SY5Y DA neurons. Collectively, these data indicate that astrocyte Panx 1 may be a key element for inflammatory response regulation in the context of PD-like pathology. Hopefully, future studies on the role of Panx 1 in neurotrophic molecule release will help us to understand the contributions of PD pathological regulation and to establish a therapeutic intervention against PD.

## Materials and Methods

### Animals and Preparation of an Acute MPTP Mouse Model

All animal procedures performed in our studies were reviewed and approved by the Animal Ethics Committee of University of Nantong, China.

1-methyl-4-phenyl-1,2,3,6-tetrahydropyridine regimen were performed according to previously described (Meng et al., 2019). Eight male mice on 8–10 weeks old were randomly divided into two groups. Mice (*n* = 4) were given three i.p. injection of 20 mg/kg MPTP (M 0896, Sigma-Aldrich, St. Louis, MO, United States) at 2 h intervals. The control mice (*n* = 4) were injected with the same volume of PBS. Two group mice were sacrificed at 4 days after injection.

### Immunofluorescence

Immunostaining protocols were performed as previously described (Chen et al., 2014). Mice were anesthetized under isoflurane and 40 μm brain slices were collected with a cryostat after transcardial perfusion with 4% paraformaldehyde. After permeabilizing with 0.3% Triton X-100 for 10 min, and blocking with 10% BSA for 2 h, slices were incubated with the following primary antibodies: GFAP (mouse; 1:500, Millipore, Billerica, MA, United States), Panx 1 (1:300; rabbit; Abcam, Cambridge, MA, United States), TH (1:2000, chicken, Abcam).

After washing, sections were incubated with the corresponding secondary antibodies: anti-mouse 488, anti-rabbit Cy3, and anti-chicken 647 (1:1000, Jackson ImmunoResearch, West Grove, PA, United States) for 2 h. Samples were mounted with anti-fade mounting medium containing Hoechst 33342, and imaged with a Nikon fluorescence microscope (Tokyo, Japan). The quantitative analysis of immunostaining images was performed using ImageJ software (Bethesda, MD, United States). The co-localization of GFAP and Panx 1 was determined by measuring the Manders’ coefficient (fraction of GFAP overlapping Panx 1). Values of these coefficients express the ratio of two fraction overlapping (McDonald and Dunn, 2013).

### Primary Astrocyte Isolation and SH-SY5Y Cell Cultures

Primary astrocyte isolation and collection were prepared as previously published with minor modification (Wei et al., 2018). Briefly, cerebral cortex was removed from 1-day-old institute of cancer research (ICR) mice. After digesting with 0.25% trypsin-EDTA solution for 15 min at 37°C, the cell pellets were collected and re-suspended in astrocyte cultured medium (DMEM + 10% FBS + penicillin/streptomycin). The cells were plated in 75 cm^2^ flasks coated with PDL (100 μg/ml) and maintained in astrocyte cultured medium for 12–14 days with medium changes every 4 days. To purify cultured astrocytes, the flasks were shaken at 250 rpm and 37°C for 16 h. The remaining attached cells were collected and identified with glial fibrillary acidic protein (GFAP), a specific marker for astrocytes, and used for subsequent study.

SH-SY5Y cells (Cell Bank of Chinese Academy of Sciences, Shanghai, China) were cultured and maintained in the same medium with astrocytes.

### RNA Isolation and qPCR Assay

Total RNA was isolated from astrocytes with RNA quick purification kit (Yishan Biotech, Shanghai, China) and reversed using the PrimeScript RT kit (RR037A, TaKaRa, Dalian, China) according to the manufacturer’s instructions. qPCR assay was performed using SYBR Green PCR Master Mix (1725260, Bio-Rad, Hercules, CA, United States). The relative levels of target mRNA were normalized to the endogenous control (glyceraldehyde-3-phosphate dehydrogenase, GAPDH or 18S) by 2^–ΔΔ*CT*^ formula. Primers used in the experiments were listed in Table 1.

**TABLE 1:** qPCR primers used in our study.

**Gene**	**Sense sequence**	**Anti-sense sequence**
Panx l	CCTCATTAACCTCATTGTGTAT	CATTGTAGCCTTCAGACTTG
Panx 2	TGTGGTCTATACTCGCTATG	CTCCTGCTGGATGTCTAG
Panx 3	CTCAGATTATGGACTATGAACAC	TCAGAAGGTAACTTGGAGAAT
18S	GACAGGATTGACAGATTGATAG	CGTTATCGGAATTAACCAGAC
IL-1β	TGTCTTGGCCGAGGACTAAG	TGGGCTGGACTGTTTCTAATG
IL-6	TCCATCCAGTTGCCTTCTTGG	CCACGATTTCCCAGAGAACATG
IL-10	CTGGACAACATACTGCTAAC	AAATGCTCCTTGATTTCTGG
TNF-α	CCCCAAAGGGATGAGAAGTT	CACTTGGTGGTTTGCTACGA
TGF-β	CCTATTTAAGAACACCCACTTT	TCCTGAATAATTTGAGGTTGAG
Caspase 3	TGGGCACATCTTCAGAAA	GTGGTAACTTGGACATCATC
		

### Western Blotting

Proteins were extracted from adult mouse brain and cultured astrocytes with a RIPA lysis buffer containing 1 mg/ml of protease inhibitors (1:100; Roche, Basel, Switzerland), and measured with a BCA kit (P0010, Beyotime, China). An equal amount of isolated total cell proteins from each group were separated by 10% SDS gel after boiling for 10 min at 95°C, and then transferred to 0.22 μm PVDF membrane, and blocked in non-fat 5% milk. The membranes were probed with the primary antibodies against Panx 1 (1:1000; rabbit; Abcam, Cambridge, MA, United States), and GAPDH (1:1000; mouse; Proteintech, Chicago, IL, United States), and then with anti-rabbit/mouse HRP-conjugated secondary antibodies (1:10000; Jackson ImmunoResearch, West Grove, PA, United States). Chemiluminescence was examined in a Bio-Rad ChemDoc (Hercules, CA, United States) with an enhanced chemiluminescence solution (Thermo Fisher Scientific, Waltham, MA, United States). The intensity of bands was analyzed by ImageJ software (National Institute of Health, Bethesda, MA, United States).

### Transfection of Panx 1 siRNA in Cultured Primary Astrocytes

Small interfering RNA (siRNA) were transferred into astrocytes with RNAiMAX (Invitrogen, Carlsbad, CA, United States) at 2 days after plating, and scrambled siRNA was used as a control. The Panx 1-targeting and scrambled siRNA were provided by RiboBio (Guangzhou, China). Panx 1 siRNA sequences used in this study are listed in Table 2.

**TABLE 2:** Panx 1-targeting siRNA sequences.

**Name**	**Target sequence**
Panx 1 siRNA1	ACAAGATGGTCACATGTAT
Panx 1 siRNA2	ACCCAATCGTGGAGCAGTA
Panx 1 siRNA3	GCCTCATTAACCTCATTGT

### Detection of IL-1β, IL-6, and IL-10 by ELISA

Astrocytes (2 × 10^5^ cells/well) were seeded in six well plates and cultured for 24 h. Cells were pretreated with siRNA for 36 h, or CBX (100 μM) for 1 h. LPS (100 ng/ml) was then added and incubated for further 24 h. Cells from different groups were collected with a RIPA lysis buffer containing 1 mg/ml of protease inhibitors (1:100; Roche, Basel, Switzerland), and measured with a BCA kit (P0010, Beyotime, China). For each reaction in a 96-well plate, 30–40 μg of proteins was used. Mouse IL-1β, IL-6, and IL-10 were measured by ELISA kit (FCMACS, Nanjing, China) according to the instructions of the manufacturer.

### Treatments and Preparation of Astrocyte Conditioned Medium (ACM)

Astrocytes were seeded in six-well plates at a density of 4 × 10^5^ and cultured for 24 h following by serum starvation for 2 h, and treated with LPS (100, 250, and 500 ng/ml; L2630, Sigma) or vehicle, as a control for 24 h. Different concentration of Panx 1 inhibitor carbenoxolone (CBX; Sigma) was pretreated for 1 h before LPS treatment at 37°C. Following with centrifugation at 12,000 rpm for 10 min at 4°C, the medium were collected as astrocyte conditioned medium with or without LPS stimulation (ACM, or ACM/LPS), or pre-treatment with CBX or siRNA (ACM/LPS + CBX; ACM/LPS + siRNA), respectively (Figure 7A), and were stored at −80°C before use.

### CCK8 Assay

SH-SY5Y cells were seeded in 96-well plates at 1 × 10^4^ per well. After treatment with different astrocyte conditioned medium or CBX alone, the cell viability was determined with a CCK8 cell counting kit (CCK-8, Dojindo, Kumamoto, Japan) according to the manufactures’ instructions. The resulting formazan crystals were measured at 450 nm. The data were presented as the ratio of the control level.

### Statistical Analysis

Data are presented as the mean ± standard error of the mean (SEM) and n indicates to the number of independent experiments. Detailed statistic results were shown in the figure legends. Data were analyzed by one-way analysis of variation (ANOVA) followed by Newman–Keuls Multiple Comparison Test for multiple comparisons and Student’s *t*-test for two groups using GraphPad Prism 5 (GraphPad Software, La Jolla, CA, United States). A *p*-value < 0.05 was considered statistically significant.

## Data Availability Statement

The raw data supporting the conclusions of this article will be made available by the authors, without undue reservation.

## Ethics Statement

The animal study was reviewed and approved by the Animal Ethics Committee of University of Nantong, China.

## Author Contributions

Z-YW and M-HC designed the project. Z-YW, Z-ML, QW, YM, PX, and YG performed the experiments and analyzed the data. Z-YW and Z-ML wrote the manuscript and supervised all aspects of the project. All authors read and approved the final manuscript.

## Conflict of Interest

The authors declare that the research was conducted in the absence of any commercial or financial relationships that could be construed as a potential conflict of interest.

## Publisher’s Note

All claims expressed in this article are solely those of the authors and do not necessarily represent those of their affiliated organizations, or those of the publisher, the editors and the reviewers. Any product that may be evaluated in this article, or claim that may be made by its manufacturer, is not guaranteed or endorsed by the publisher.

## Supplementary Material

Supplementary Figure 1 **(A)** Immunostaining with TH (red) in SN and with GFAP (green) in striatum after injection with PBS or MPTP on 4 days. **(B)** Quantification of TH + neurons in PBS and MPTP groups. Statistical comparison were performed using Student’s *t*-test; ^∗∗^*p* < 0.01. Scale bar, 20 μm.

Supplementary Figure 2 **(A,B)** The effect of MPP^+^ at different concentrations for 24 h on the viability of SH-SY5Y cells and primary cultured astrocytes. The data were normalized to control group (0 μM MPP^+^) levels, *n* = 3. All data are mean ± SEM. Statistical comparison were performed using one-way ANOVA, following by Newman–Keuls Multiple Comparison Test. ^∗∗∗^*p* < 0.001 compared with the control group.

Supplementary Figure 3 The influence of MPP^+^ at different concentrations for 24 h on the expression of selected inflammatory cytokines, IL-6 **(A)**, IL-1β **(B)**, and TGF-β **(C)** in primary cultured astrocytes. The data were normalized to the control group (0 μM MPP^+^) levels, *n* = 3. All data are mean ± SEM. Statistical comparison were performed using one-way ANOVA, following by Newman–Keuls Multiple Comparison Test. ^∗^*p* < 0.05, ^∗∗^*p* < 0.01, ^∗∗∗^*p* < 0.001 compared with the control group.

Supplementary Figure 4 The effect of Panx 1-targeting siRNA on Panx 2 and Panx 3 expression in cultured primary astrocytes. The data were normalized to the scramble siRNA group level.

Supplementary Figure 5 qPCR analysis of IL-1β, IL-6, and TNF-α expression after treatment with scrambled or Panx 1-targeted siRNA alone in primary cultured astrocytes. The data were normalized to control group levels.

Supplementary Figure 6 qPCR analysis of Panx 2 and Panx 3 expression after 100 ng/ml LPS treatment in primary cultured astrocytes. The data were normalized to control group levels.

Supplementary Figure 7 Analysis of the selected inflammatory cytokine mRNA expression by qPCR assay, including IL-1β **(A)**, IL-6 **(B)**, TNF-α **(C)**, TGF-β **(D)**, and IL-10 **(E)** in primary cultured astrocytes. Cells were pretreated with Panx 1 inhibitor CBX (100 mM, 60 min) alone or LPS treatment (100 ng/ml, 24 h). The levels were displayed as percentage of the control (the same volume of DMSO). The data were normalized to 18S mRNA levels, *n* = 3. All data are mean ± SEM. Statistical comparison were performed using one-way ANOVA, following by Newman–Keuls Multiple Comparison Test. ^∗^*p* < 0.05, ^∗∗∗^*p* < 0.001 compared with the control group.

## Abbreviations

Panx 1: Pannexin 1
MPP^+^: 1-methyl-4-phenylpyridinium
CBX: carbenoxolone
SN: substantia nigra
IL-1β: interleukin-1 beta
IL-6: interleukin-6
CX 43: connexin 43
GFAP: glial fibrillary acidic protein
TNF-α: tumor necrosis factor α
IL-10: interleukin-10
TGF-β: transforming growth factor-β
FGF1: fibroblast growth factor 1.
