# Supplementary figures and images for: Astrocyte Pannexin 1 Suppresses LPS-Induced Inflammatory Responses to Protect Neuronal SH-SY5Y Cells
Source: Front Cell Neurosci. 2021 Aug 12;15:710820. doi: 10.3389/fncel.2021.710820 (PMC8406772; doi:10.3389/fncel.2021.710820)

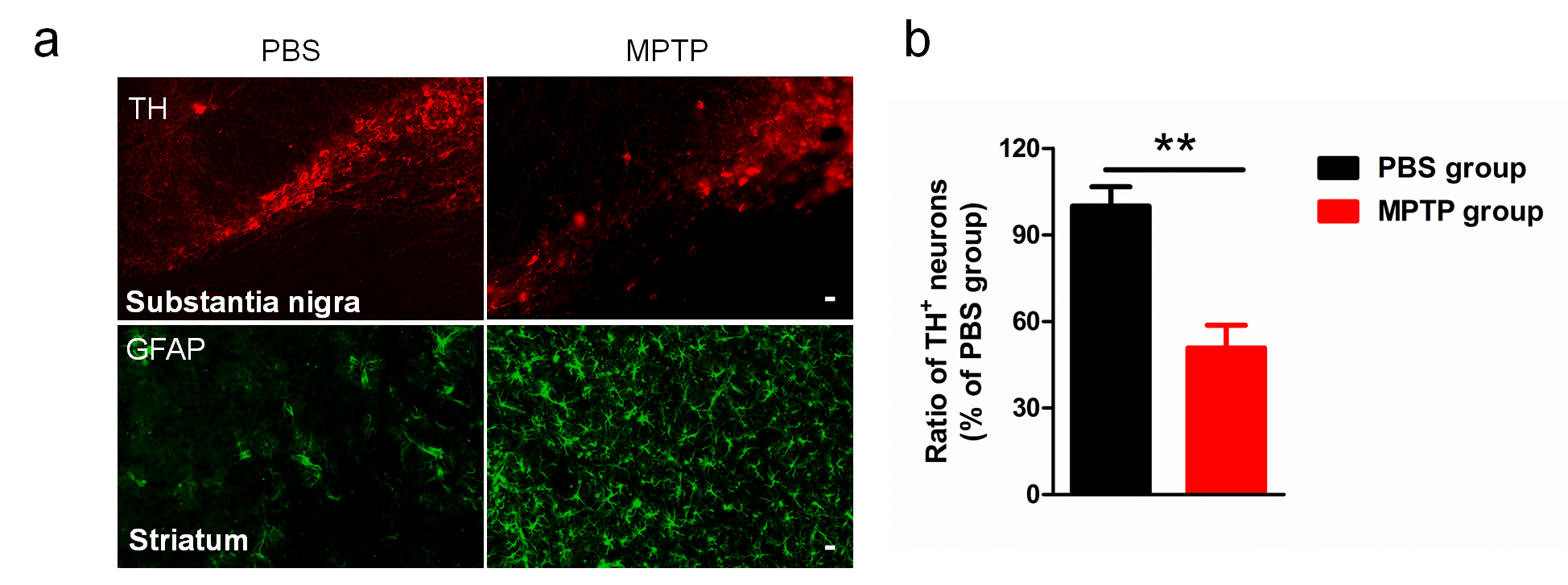

Supplement: Supplementary Figure 1 — (A) Immunostaining with TH (red) in SN and with GFAP (green) in striatum after injection with PBS or MPTP on 4 days. (B) Quantification of TH + neurons in PBS and MPTP groups. Statistical comparison were performed using Student’s t-test; ∗∗p < 0.01. Scale bar, 20 μm. [file Image_1.TIF]

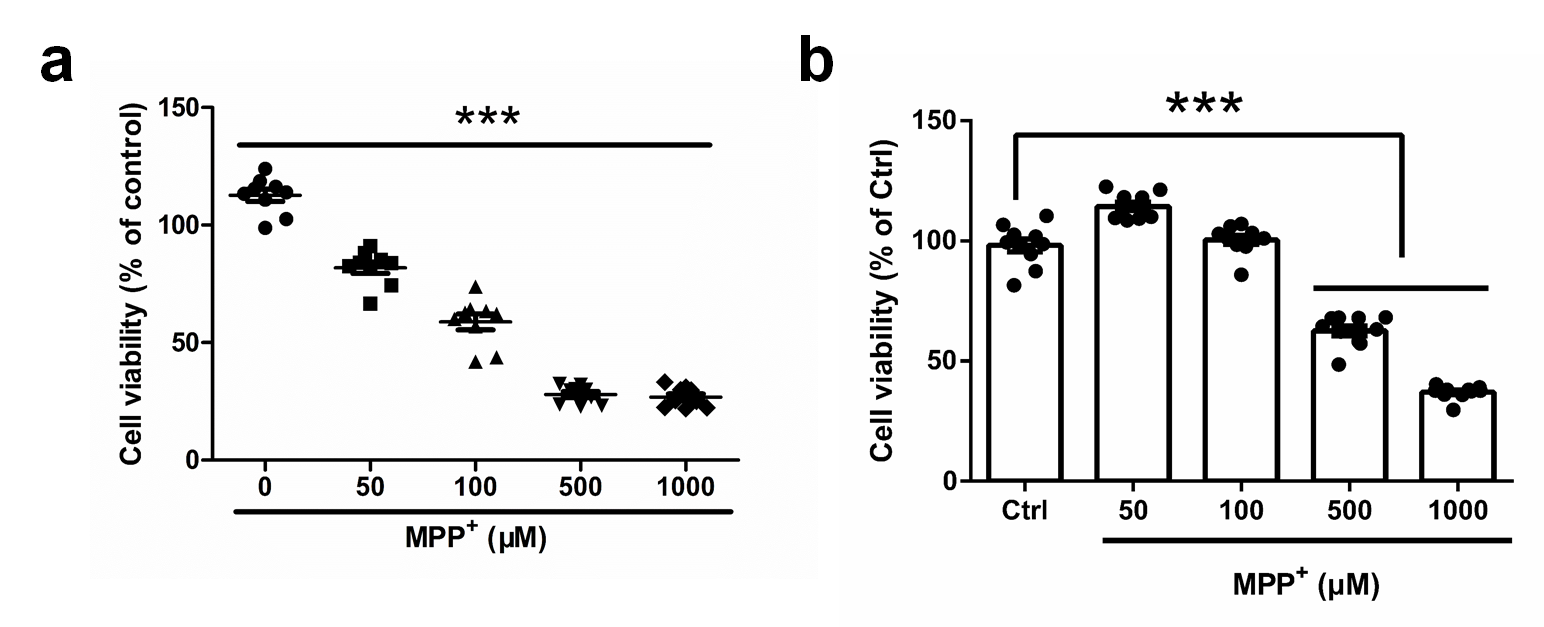

Supplement: Supplementary Figure 2 — (A,B) The effect of MPP+ at different concentrations for 24 h on the viability of SH-SY5Y cells and primary cultured astrocytes. The data were normalized to control group (0 μM MPP+) levels, n = 3. All data are mean ± SEM. Statistical comparison were performed using one-way ANOVA, following by Newman–Keuls Multiple Comparison Test. ∗∗∗p < 0.001 compared with the control group. [file Image_2.tif]

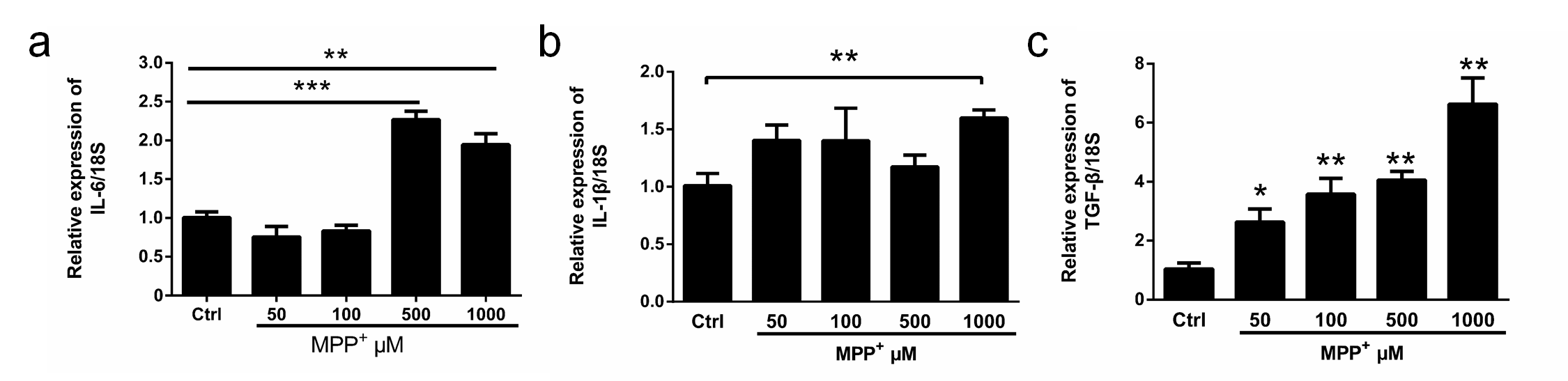

Supplement: Supplementary Figure 3 — The influence of MPP+ at different concentrations for 24 h on the expression of selected inflammatory cytokines, IL-6 (A), IL-1β (B), and TGF-β (C) in primary cultured astrocytes. The data were normalized to the control group (0 μM MPP+) levels, n = 3. All data are mean ± SEM. Statistical comparison were performed using one-way ANOVA, following by Newman–Keuls Multiple Comparison Test. ∗p < 0.05, ∗∗p < 0.01, ∗∗∗p < 0.001 compared with the control group. [file Image_3.TIF]

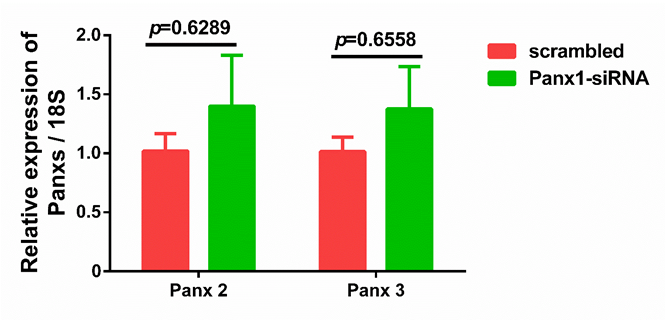

Supplement: Supplementary Figure 4 — The effect of Panx 1-targeting siRNA on Panx 2 and Panx 3 expression in cultured primary astrocytes. The data were normalized to the scramble siRNA group level. [file Image_4.TIF]

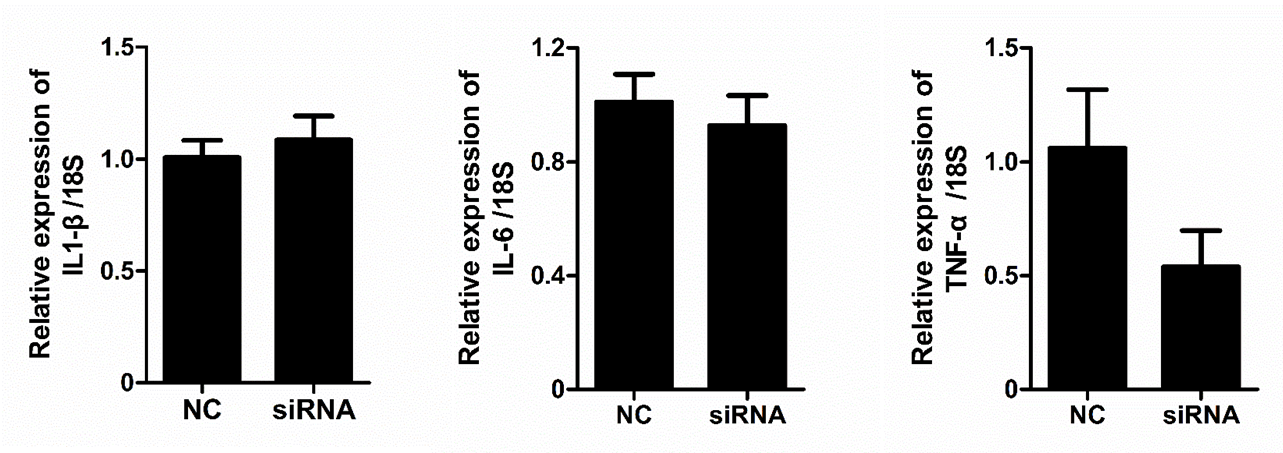

Supplement: Supplementary Figure 5 — qPCR analysis of IL-1β, IL-6, and TNF-α expression after treatment with scrambled or Panx 1-targeted siRNA alone in primary cultured astrocytes. The data were normalized to control group levels. [file Image_5.TIF]

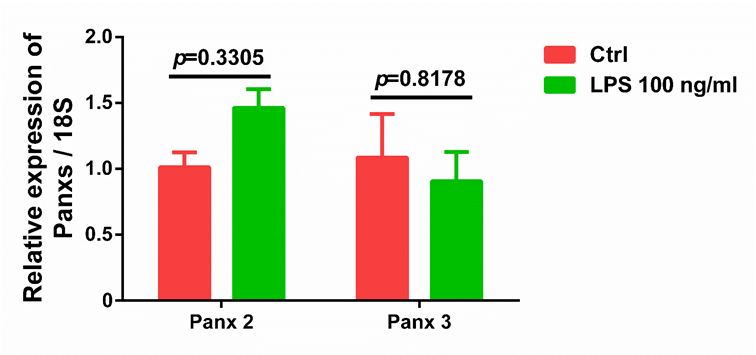

Supplement: Supplementary Figure 6 — qPCR analysis of Panx 2 and Panx 3 expression after 100 ng/ml LPS treatment in primary cultured astrocytes. The data were normalized to control group levels. [file Image_6.TIF]

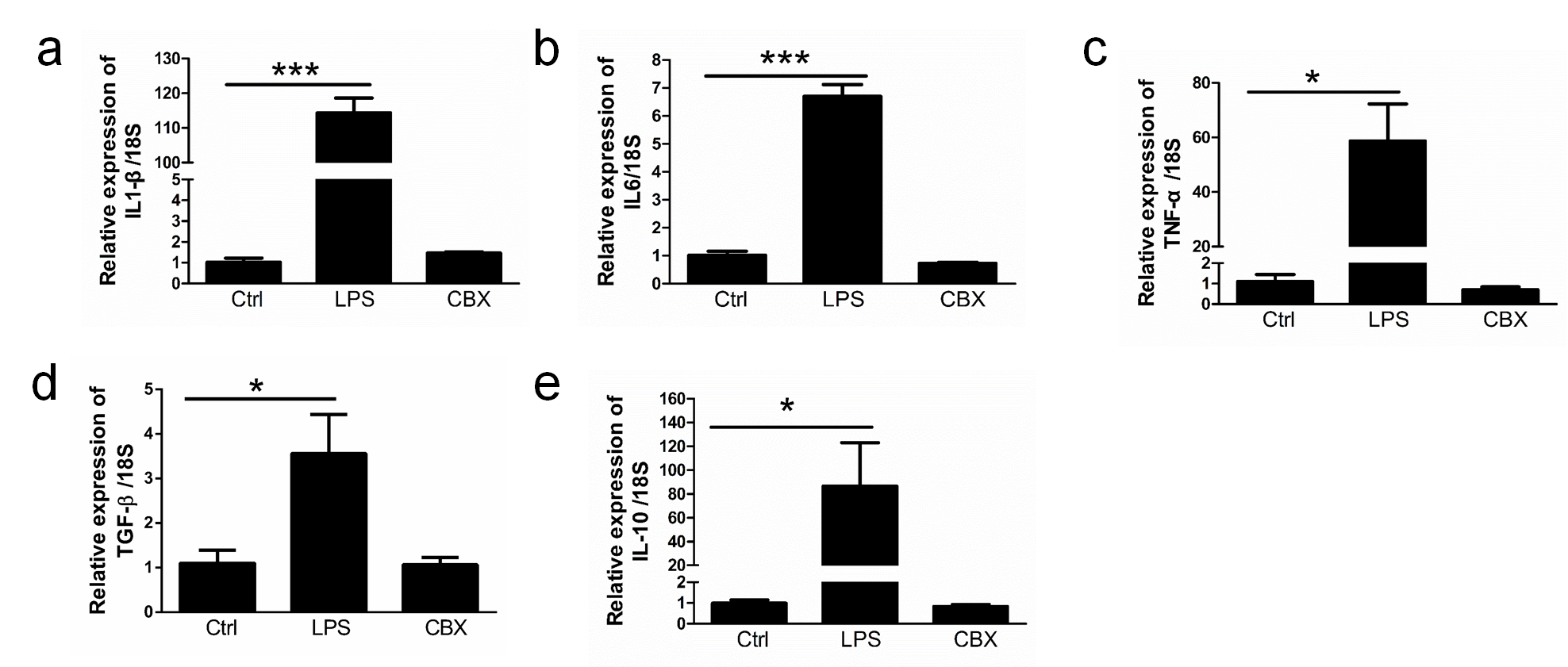

Supplement: Supplementary Figure 7 — Analysis of the selected inflammatory cytokine mRNA expression by qPCR assay, including IL-1β (A), IL-6 (B), TNF-α (C), TGF-β (D), and IL-10 (E) in primary cultured astrocytes. Cells were pretreated with Panx 1 inhibitor CBX (100 mM, 60 min) alone or LPS treatment (100 ng/ml, 24 h). The levels were displayed as percentage of the control (the same volume of DMSO). The data were normalized to 18S mRNA levels, n = 3. All data are mean ± SEM. Statistical comparison were performed using one-way ANOVA, following by Newman–Keuls Multiple Comparison Test. ∗p < 0.05, ∗∗∗p < 0.001 compared with the control group. [file Image_7.TIF]
